# Monitoring Vital Signs: Development of a Modified Early Warning Scoring (Mews) System for General Wards in a Developing Country

**DOI:** 10.1371/journal.pone.0087073

**Published:** 2014-01-24

**Authors:** Una Kyriacos, Jennifer Jelsma, Michael James, Sue Jordan

**Affiliations:** 1 Division of Nursing and Midwifery, Department of Health & Rehabilitation Sciences, Faculty of Health Sciences, University of Cape Town, South Africa; 2 Department of Health and Rehabilitation Sciences, Faculty of Health Sciences, University of Cape Town, South Africa; 3 Department of Anaesthesiology, Groote Schuur Hospital/University of Cape Town, South Africa; 4 School of Human and Health Sciences, Swansea University, Wales, United Kingdom; D’or Institute of Research and Education, Brazil

## Abstract

**Objective:**

The aim of the study was to develop and validate, by consensus, the construct and content of an observations chart for nurses incorporating a modified early warning scoring (MEWS) system for physiological parameters to be used for bedside monitoring on general wards in a public hospital in South Africa.

**Methods:**

Delphi and modified face-to-face nominal group consensus methods were used to develop and validate a prototype observations chart that incorporated an existing UK MEWS. This informed the development of the Cape Town ward MEWS chart.

**Participants:**

One specialist anaesthesiologist, one emergency medicine specialist, two critical care nurses and eight senior ward nurses with expertise in bedside monitoring (N = 12) were purposively sampled for consensus development of the MEWS. One general surgeon declined and one neurosurgeon replaced the emergency medicine specialist in the final round.

**Results:**

Five consensus rounds achieved ≥70% agreement for cut points in five of seven physiological parameters respiratory and heart rates, systolic BP, temperature and urine output. For conscious level and oxygen saturation a relaxed rule of <70% agreement was applied. A reporting algorithm was established and incorporated in the MEWS chart representing decision rules determining the degree of urgency. Parameters and cut points differed from those in MEWS used in developed countries.

**Conclusions:**

A MEWS for developing countries should record at least seven parameters. Experts from developing countries are best placed to stipulate cut points in physiological parameters. Further research is needed to explore the ability of the MEWS chart to identify physiological and clinical deterioration.

## Introduction

### Background

In well-resourced settings, observations charts often incorporate early warning or modified early warning scoring (EWS/MEWS) systems [1]. These are bedside score and track-and-trigger systems: nurses score observations of vital signs and calculate a total score to facilitate early recognition of a patient’s deterioration. EWS/MEWS systems are used in conjunction with nurses’ clinical judgement.

Abnormal physiology is common on general hospital wards [2]; clinical and physiological deterioration is evident for six [3] to eight hours [4] before cardiopulmonary arrest. In such cases, arrest often occurs after a period of slow and progressive physiological deterioration that went unrecognized and/or inadequately treated hypoxaemia and hypotension [5]. Non-recognition of deterioration in clinical status has implications for patient survival, which depends on nurses’ decisions to summon assistance. Clinical signs such as skin tone, sweating, nausea or nurses’ intuitive assessment of the patient being ‘just not right’ and ‘looking unwell’ [6] should be monitored regularly to limit avoidable, serious adverse events (SAEs) such as cardiac arrest, urgent and unanticipated admission to an intensive care unit (ICU) or even death. In addition to obvious ethical considerations, authorities in the developed world are concerned at the increasing number of claims for malpractice associated with SAEs [7].

### Developing MEWS Charts

In 1997 Morgan, Williams and Wright [8] in the UK were the first to develop and publish the EWS of five physiological parameters (heart rate, systolic blood pressure, respiratory rate, temperature and conscious level). Each parameter had a range of cut points with corresponding colour-banded trigger points (scores) (0, upper and lower 1 to 3) not to predict outcome [9] but to serve as a track-and-trigger system (TTS) to identify early signs of deterioration [10]. For example a heart rate cut point range of 111–129 bpm is awarded a trigger point of 2 indicating the need for escalation of intervention.

Since then EWS systems have been modified (MEWS) and standardized (SEWS) [11], [12] across the UK [13]. In addition to the original five physiological parameters included in most EWS [14], [15], oxygen saturation [16], [17] and urine output [18] are included in some EWS observations charts. In the UK urine output is incorporated in the chart but not scored [13], [19]. Clinical signs of deterioration (pallor, sweating, looking unwell) [20] are incorporated into MEWS charts although these are not scored. Of 23 aggregate weighted track and trigger systems, only one incorporated ‘nurse concern’ [21].

A multiplicity of EWS systems in the UK resulted in lack of consistency in the recognition of and response to clinical deterioration, necessitating a standardized national early warning system (NEWS) [13]. Lack of standardization of assessment, monitoring and tracking of clinical deterioration may have meant that critical care outreach teams in the UK [22] and Australia [23] were not used optimally. Implementation of a National EWS (NEWS) in July 2012 in the UK for monitoring six parameters (respiratory rate, oxygen saturations, temperature, systolic BP, heart rate and level of consciousness) is not mandatory in all hospitals but is advocated to improve patient outcomes [13].

Vital signs charts used in public hospital wards in Cape Town and the wider Western Cape Province do not incorporate ‘track’ and ‘trigger’ algorithms (Figure S1: Example of an existing observations chart used in research setting). Physiological readings are plotted graphically on the chart using symbols (x for BP, • for pulse), often intersecting and impeding visibility. At the research setting there were no hospital-wide emergency response systems, no ‘calling criteria’ (triggers) with predefined thresholds for physiological parameters and no early warning scoring systems on general wards outside critical care areas. Therefore, we were concerned that early warning signs of physiological deterioration might go undetected and that without a reporting algorithm rescue interventions might lack consistency: this would have the potential to compromise patient safety and clinical outcomes.

**Figure 1:**
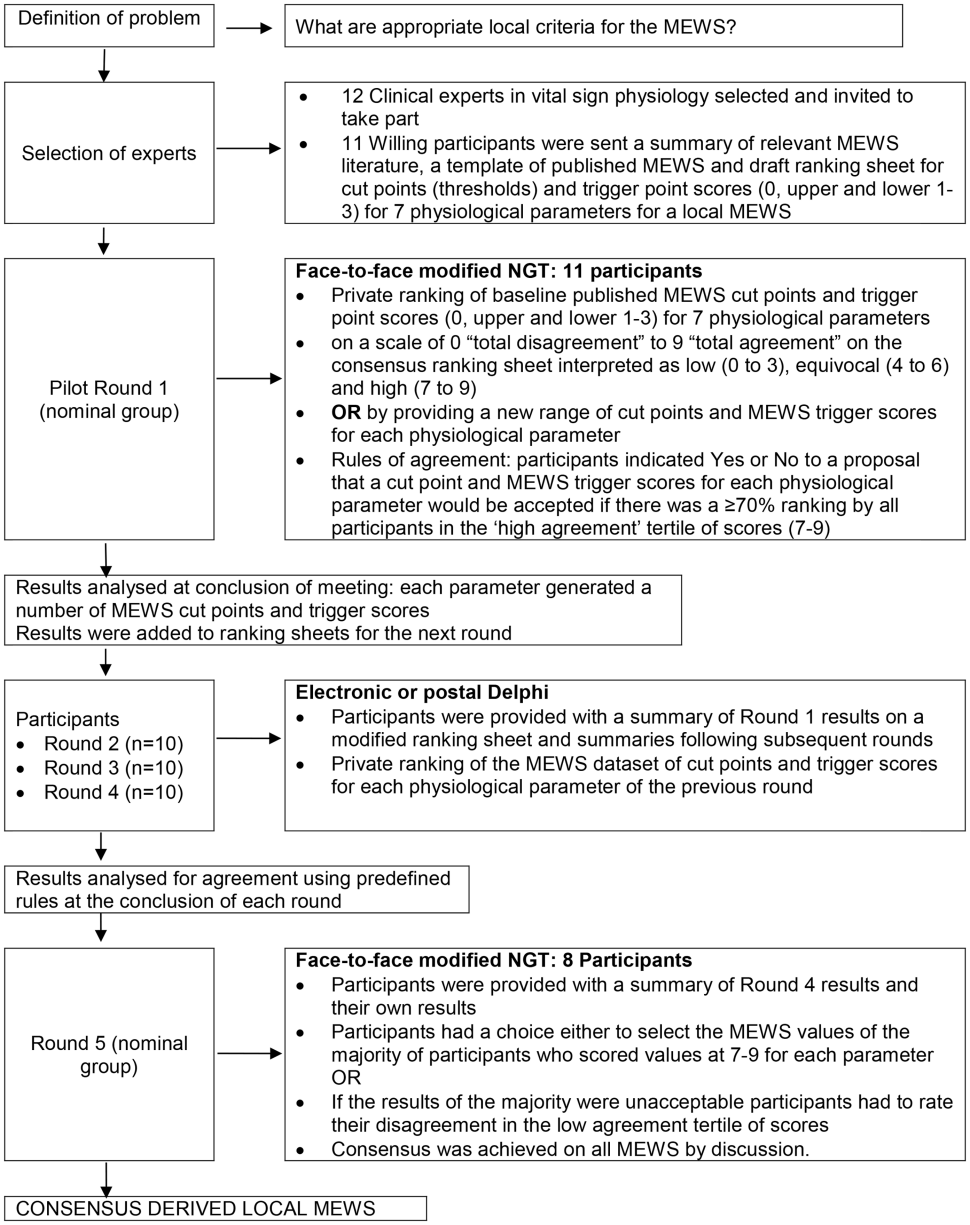
Procedure for consensus methods.

**Figure 2:**
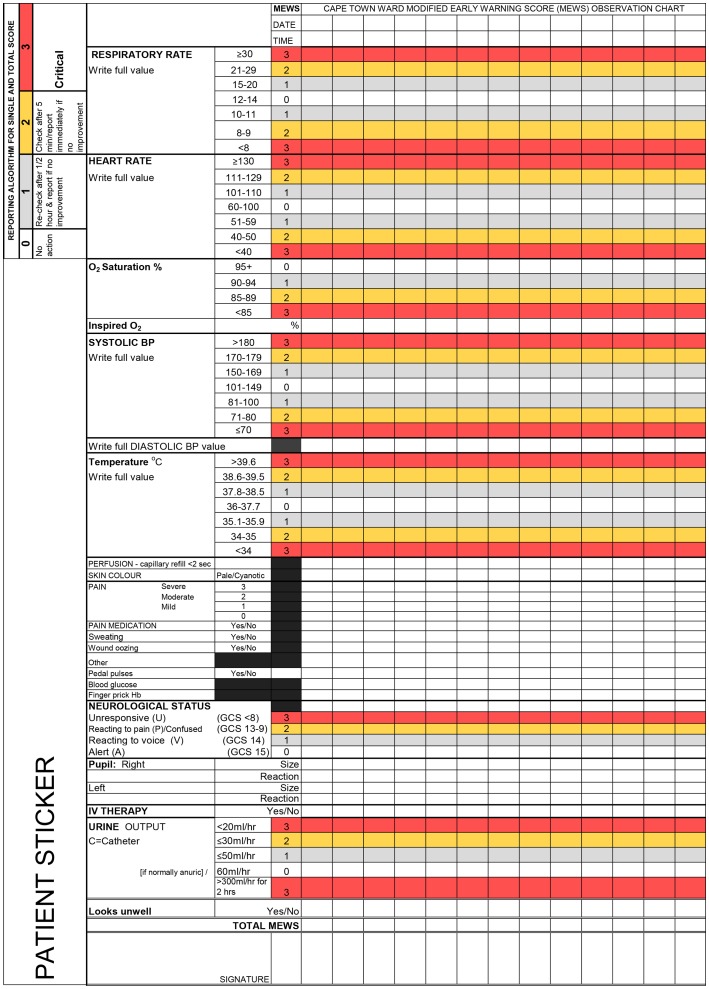
Consensus derived Cape Town ward MEWS observations chart and reporting algorithm.

The ideal MEWS does not exist. For this reason consensus methods were appropriate for reaching agreement about local criteria for the Cape Town ward MEWS. This paper describes how the published evidence on track-and-trigger early warning scoring (EWS) systems and consensus methods (Figure 1: Procedure for consensus methods [24]) were used to develop a preliminary MEWS chart (Figure 2: Consensus derived Cape Town ward MEWS observations chart and reporting algorithm) for use on general surgical hospital wards. General wards have fewer nurses to look after higher numbers of patients. These patients are judged not to need close observation and one-to-one care, unlike patients in intensive care and high dependency units.

## Methods

### Design

Delphi and nominal group consensus methods were employed for a multidisciplinary approach to derive a contextually suitable MEWS from an existing UK MEWS and to develop a prototype observations chart that incorporated the MEWS.

### Research Setting

The study was conducted over four months in 2009 in a single centre research setting: a 867-bed academic public (government) hospital in Cape Town purposively selected from two such hospitals because English was the dominant language.

### MEWS Construction

#### Physiological parameters incorporated

We searched the published literature for MEWS cut points (thresholds) and trigger points (scores). A MEWS table (Table 1) was constructed and comprised seven physiological parameters (respiratory rate, heart rate, systolic BP, temperature, level of consciousness [15], oxygen saturation (SaO2) [12] and urine output [25].

**Table 1:** Prototype Modified Early Warning Scoring System constructed from the literature.

	3	2	1	0	1	2	3
Respiratory rate/min		9 or less		9–14	15–20	21–29	30 or more
SaO_2_	<85	85–89	90–92	93+			
Heart rate/min		40 or less	41–50	51–100	101–110	111–129	130 or more
BP systolic	70 or less	71–80	81–100	101–199		200 or more	
Temperature ^o^C		35 or less		35–38.4		38.5 or more	
NEUROLOGICAL STATUS Glasgow Coma Scale (GCS)				15	14 Change in mentation	GCS 13–9	GCS ≤8 or unresponsive
OR AVPU				Alert	Reacting to voice	Reacting to pain	Unresponsive
Urine mls/kg/hr	0.5 ml/kg/1 hr or less		1 ml/kg/1 hr or less	If normally anuric score 0	3 ml/kg/1 hr or more		

Aggregated score = GCS 15 = A; GCS 14 = V; GCS 13–9 = P; GCS ≤8 = U.

Interpretation: Aggregated MEWS: 3 = critical score.

(Adapted from Subbe, Kruger et al., 2001 [15]; Morrice and Simpson, 2007 [25], Paterson, MacLeod et al., 2006 [12]).

#### Scoring system and reporting algorithm

For each physiological parameter a range of MEWS cut points (thresholds) was defined. Each variable was partitioned for the recording of actual readings (eg. respiratory rate of 21). For each threshold a weighted trigger score of an upper or lower 1 to 3 was assigned and colour- coded, indicating the magnitude of deviation from the ’normal’ range (defined as 0). Both single parameter and multiple-parameter track-and-trigger systems were incorporated so that a reporting algorithm would be followed both for a total (aggregated) score outside the normal range AND any one single parameter (a ‘combination’ system) [26].

The reporting algorithm gave decision rules to determine the urgency level [27]. The rules were: 0 = no action; 1 = re-check after 30 minutes and report if no improvement; 2 = check after 5 minutes/report immediately if no improvement; 3 = critical REPORT IMMEDIATELY.

#### Prototype MEWS observations chart

The construction of the MEWS table was followed by a search for an observations chart that not only incorporated a MEWS but also criteria for clinical signs of deterioration. Permission was requested to adapt charts with clinical indicators for South Africa. A preliminary prototype MEWS observations chart was designed. Clinical indicators from published and local existing charts were included in the MEWS prototype chart to contribute to recognition of deterioration: heart rhythm [28], inspired oxygen %, diastolic blood pressure, perfusion (capillary refill), pallor/cyanosis and ‘looks unwell’ [20], subjective pain (scored 0–3 severe by the patient), sweating, administration of pain medication, wound oozing, jaw wired (yes/no), abdominal girth, pedal pulses, blood glucose, finger prick Hb, pupil size and intravenous therapy.

### Participants

Inclusion criteria for participants, sampling methods and sample size for the consensus methods are summarised in Table 2. The 12 potential participants were invited by e-mail and/or arranged interview to a one-hour face-to-face consensus development workshop in a conference facility in the research setting. A neurosurgeon was invited to all rounds as the MEWS advocated replacement of the Glasgow Coma Scale (in use at the setting) with the AVPU (Alert/Responding to Voice/Responding to Pain/Unresponsive) system. However, when the emergency medicine specialist was unavailable the neurosurgeon substituted so was not counted as an additional member. Participants were selected for their expertise in the clinical area where the MEWS was to be deployed – adult surgical wards caring for patients in the post-operative period.

**Table 2:** Participants and sampling methods for consensus development of the MEWS chart.

Research activity	Sampling method	Inclusion criteria	Respondents/Participants (n = 12*)	Rationale
Consensusdevelopment ofthe MEWS	Purposivesampling	Medical experts in clinicalphysiology and health sciencesresearch (including CCNs)and senior	1 PhD specialist anaesthesiologist 1 PhD emergencymedicine specialist with experience in implementinga triage early warning scoring (TEWS) inCape Town	A mixed panel of expertsrepresents the diversity foundon a ward who are all
		ward nurses with expertise in	2 Critical care nurses/lecturers with a Master’s degree	involved in bedside
		bedside monitoring	6 ‘head’ nurses – I from each of the researchsurgical wards;	monitoring to some extent
			1 surgical nurse operational manager¥	
			1 surgical nurse clinical educator	
			*1 PhD neurosurgeon	

Note on table:

* The neurosurgeon who had been fully informed of the study from its inception was included as a replacement for the emergency medicine specialist in Round 5.

¥ The surgical nurse clinical educator participated in all consensus rounds but replaced one surgical head nurse after Round 1.

The Head of the Department of Surgery was fully informed of the study from its inception but was not available to participate in the consensus rounds.

### Procedure for Seeking Consensus

The literature on consensus methods for solving problems in health care and particularly for the derivation and validation of MEWS is summarized in Table 3. MEWS reading material was distributed to consensus members before the meeting. The procedures employed during consensus Round 1 to the final Round 5 and the number of participants, are summarized in Figure 1.

**Table 3:** Summary of the literature on consensus methods for solving problems in health care.

Consensus method	Characteristics/Advantages	Disadvantages
Delphi first introduced in 1948 [37]	Uses expert panels.	Members drop out often from fatigue [37].
	Requires surveys by questionnaire and/or electroniccommunication (e-mail) for multiple rounds.	Decisions are limited by group members and their pastexperience or work in the field [32].
	Inexpensive data collection method, relyingon repeated rounds of commentsfrom experts.	Criticized for being less representative than the RAND-UCLAappropriateness multidisciplinary panels [52].
	Reliability increases with the size of the group and thenumber of rounds [37].	There is the potential for bias [32] and not having inter-raterreliability testing [31].
	After each round data are analysed and collated into onedocument in preparation for the next round [32].	Is generally inferior to the nominal group technique, albeitto a small degree [24].
	The outcome is a combined opinion achieved in astructured and anonymous way [32].	Difficulties relate to practical rather than theoreticalconsiderations and more research is needed to clarify theconcept expertise.
	The Delphi has been modified [31].	
Nominal Group Technique firstdescribed in 1971 by Delbecq andVan de Ven [53]	Is used to create a structured environment in whichexperts are given the best available information forconsidering solutions that are more justifiableand credible than may bethe case otherwise [37].	Face-to-face consensus methods place more responsibilityon the leader than is the case for the Delphi technique,and the NOMINAL GROUP TECHNIQUE therefore requires objectiveand skilled leaders [37].
	Is used for obtaining consensus in an orderly manner frompersons closely associated with a problem area, andis based on the National Institutes of Health (NIH) andthe Glaser approach to consensus [37].	Jones and Hunter (1995) modified the NOMINAL GROUPTECHNIQUE by having a different mix of participants infurther rounds as there is a potential for bias in theselection of experts.
	Is useful to establish agreement on controversialsubjects [37].	
	There is no hard and fast rule about the number ofexperts to include in a nominal group but 9–12 arerecommended and lay persons can beincluded [24].	
	The modified NOMINAL GROUP TECHNIQUE is facilitated byan expert or credible non-expert while another person takesthe role of non-participant observer collecting qualitativedata from the discussion but is not concerned with analysisof the group process [24].	
Consensus conference used by theNational Institutes of Health (NIH)since 1977 [37]	Consists of expert multidisciplinary member panels andoften involves national task forces and committeesand national and international leadersin the field.	Resource intensive.
	Is useful where there is clinical uncertainty [54].	Includes pre-conference preparation of questions andanswers by experts in the field.
	Conference proceedings last from 1.5 to 2.5 daysfollowed by dissemination and evaluation ofrecommendations [54].	
RAND-UCLA appropriatenessmethod developed in 1984 by theHealth Services UtilizationStudy [55]	A systematic method combining expert multidisciplinaryclinical opinion and evidence [56].	Resource intensive.
	A rough screening test for specific medical andsurgical procedures [52].	Patient preferences are often neglected [55].
	Measures appropriateness of health services andappropriateness of health settings for quality andcost considerations [55].	There is concern about the method’s subjectivity andunreliability [52].
	Can have a 9–12 member multidisciplinary expertpanel [57].	
	Evidence of good reproducibility [52].	
	A modified RAND appropriateness model combinedcharacteristics of both the Delphi and nominal grouptechnique [58].	
	Discussion rounds can be scored using continuousinteger scales of 1–9 [57].	

#### Ranking

A ranking sheet was constructed to derive consensus amongst experts on the number of physiological parameters to include in the chart, cut points and scores. The ranking sheet comprised continuous integer scales of 0–9. A scale of 0 “total disagreement” to 9 “total agreement” [24] was defined using a predetermined 3-point scoring scale (tertile) to interpret participants’ rankings as: low (0 to 3), equivocal (4 to 6) and high (7 to 9).

The literature does not indicate when to assume achievement of consensus, but it is important to establish the level of consensus and rules of agreement in advance, to enhance the transparency and democracy of decision-making [29], [30]. Options include >70% [31] and >80% [32] agreement. We accepted consensus for inclusion as agreement at ≥70% at high tertile scores (7 to 9) [31].

The consensus group needs to establish whether strict or relaxed ‘rules’ for agreement will apply [24]. For strict rules, all agreement ratings are within a predefined 3 point region (e.g. 1–3, 4–6, 7–9) whereas for relaxed rules, ratings fall within a 3 point region but not within a predefined region. Secondly, if extreme rankings have an undue influence on the final results, all ratings for each statement are included and then one extreme high and one extreme low rating for each statement can be excluded [24]. Initially we accepted strict rules but this changed to both strict and relaxed rules in the final round.

The cut points and associated scores for each parameter of an existing published MEWS (Table 1) were inserted in the ranking sheet. Experts had a choice of either ranking the existing values or generating a new range for each parameter. Results for each round were analysed for agreement before proceeding to the next round. Verbal agreement by the majority of group members was obtained for ranking of clinical indicators, layout of parameters on the chart and the reporting algorithm.

### Ethical Considerations

The study was approved by the University of Cape Town Faculty of Health Sciences’ Human Research Ethics Committee (REC REF 192/2009), hospital and nursing management and clinical structures. Instead of the required written consent from participants, explained at the first face-to-face nominal group conference, these clinical experts (specialist neurosurgeon, anaesthetist, emergency medicine physician, critical care nurses and senior surgical nurses) indicated that their participation in the consensus processes, that included Delphi rounds, was proof of their autonomous consent. Participants were known to the researcher and therefore not anonymous, nevertheless, a unique identification code had been assigned to each participant on the consensus ranking sheet, known only to the first author (UK) and in this way consent to participate was recorded.

## Results

### Consensus Results

Of the 12 potential participants (Table 2), 11 agreed to participate. Following five rounds (two nominal groups; three Delphi) each involving between eight and 11 experts, the Cape Town ward MEWS observations chart was derived by consensus (Figure 2). The size of the Delphi group remained constant (n = 10, 90.9%) during the three rounds. Seven of the 11 (63.6%) participants contributed to all five rounds of the MEWS (the neurosurgeon replaced the emergency medicine specialist who was unavailable in Round 5); two participants (18.2%) made one verbal and three written contributions, one participant (9.1%) made two verbal and two written contributions, and one participant (9.1%) made only one verbal contribution. Workload constraints prevented the surgical nurse manager’s participation after round 1, leaving ten participants.

There was 100% agreement (within the high tertile region of 7 to 9 ranking) on inclusion of 3 of 7 published MEWS cut points for three physiological parameters (respiratory rate, heart rate and systolic blood pressure) and for 1 of 5 published MEWS cut points for urine output. There was 100% agreement (within the high tertile region of 7 to 9 ranking) on inclusion seven completely new MEWS cut points for temperature. Deviation from published MEWS is shown in Table 4 and Table S2: Comparison of trigger thresholds for published and local MEWS parameters. For the remaining two parameters (level of consciousness, 62.5% agreement on the AVPU system and oxygen saturation, 50% agreement on 2 of 4 published MEWS cut points) a relaxed rule of <70% agreement (within the high tertile region of 7 to 9 ranking) was applied. Oxygen saturation is an early sign of an impending serious adverse event (SAE) [33].

**Table 4:** A comparison of study findings to existing literature for a local set of MEWS.

Study findings	Proportion of deviation from published MEWS in template inTable 1 (% agreement by consensus)	Previous literature
Respiratory rate	4/7 cut points deviated from published MEWS [15] (100.0% agreement by ranking within the high tertile region of 7 to 9 by applying a strict rule)	Measured in all the studies on reliability and validity testing and in nine studies on performance of MEWS [1].
		Measured in all six papers included in a systematic review [41].
		Found to be the best discriminator of clinical outcomes [59].
Heart rate	4/7 cut points deviated from published MEWS [15] (100.0% agreement by ranking within the high tertile region of 7 to 9 by applying a strict rule)	Measured in all the studies on reliability and validity and in eight studies on performance of MEWS [1]
		Measured in all six papers included in a systematic review [41].
Systolic blood pressure	4/7 cut points deviated from published MEWS [15] (100.0% agreement by ranking within the high tertile region of 7 to 9 by applying a strict rule)	A systolic blood pressure of 80–100 mmHg is reportedly an early sign frequently associated with SAEs [33].
		Measured in five studies for reliability and validity and in eight studies on performance of MEWS [1]
		Measured in all six papers included in a systematic review.
Temperature	All seven cut points deviated from published MEWS [15] (100.0% agreement by ranking within the high tertile region of 7 to 9 by applying a strict rule)	The top two most effective aggregate weighted track and trigger systems able to discriminate between survivors and non-survivors incorporated temperature monitoring [21].
		Measured in five studies on reliability and validity and in seven studies on performance of MEWS [1]
		Measured in 4/6 papers included in a systematic review [41].
Urine output	4/5 cut points deviated from published MEWS unchanged [25](100.0% agreement by ranking within the high tertile region of7 to 9 by applying a strict rule)	Measured in all six papers included in a systematic review [41] but found to be missing in 97.1% of sets of observations in one of the five studies [18].
		Measured in four studies on performance of MEWS [1] and in two studies on reliability and validity testing [14], [18]
Level of consciousness	The AVPU remained unchanged from the published literature[15] (62.5% agreement by applying a relaxed rule of rankingwithin the high tertile region of 7 to 9)	Alteration in mentation is reportedly an early sign frequently associated with SAEs [33].
		Measured in five studies on reliability and validity and in eight studies on performance of MEWS listed in Kyriacos et al., 2011.
		Measured in all six papers included in a systematic review [41].
Oxygen saturation	2/4 cut points deviated from published MEWS (Subbe, Kruger,Rutherford & Gemmel, 2001) (50.0% agreement by applyinga relaxed rule of ranking within the high tertile region of7 to 9)	Oxygen saturation of 90–95% is reportedly an early sign frequently associated with SAEs [33].
		Measured in two studies on reliability and validity [16], [17].
		Measured in 2/6 papers included in a systematic review [41].
		Measured in three studies on performance of MEWS [12], [21], [60] listed in Kyriacos et al., 2011.
Clinical variables onthe chart were not tobe scored	Inspired oxygen a new addition	Adapted with permission [61]
	Perfusion a new addition	Adapted from an existing chart at the research site
	Skin pallor/cyanosis	Adapted from an existing chart at the research site
	Pain score a new addition	Adapted with permission [28]
	Pain medication	Adapted from an existing chart at the research site
	Sweating a new addition	
	Wound oozing	Adapted from an existing chart at the research site
	Pedal pulses	Adapted from an existing chart at the research site
	Blood glucose	Consensus group
	Finger prick Hb	Adapted from an existing chart at the research site
	IV therapy	Adapted from an existing chart at the research site
	‘Looks unwell’ a new addition	Adapted with permission [61]

At the final Round 5 (face-to-face meeting) the layout of parameters on the observations chart and reporting algorithm were agreed (Figure 2) with changes. To enlarge the spaces for recording vital signs’ data, the reporting algorithm and the space for patient identification data were moved from the top of the chart and presented in vertical text alongside the physiological and clinical criteria. Changes were also made to clinical indicators: heart rhythm was deleted; wiring of the jaw and abdominal girth measurements were replaced with ’other’. The final version of the consensus derived Cape Town ward MEWS chart differed in every respect from the existing chart in use at the research setting: layout, content (physiological and clinical parameters to be monitored), method of charting and function. The consensus group agreed that the reporting algorithm should be used for single parameters and for aggregated MEWS scores.

## Discussion

### Principal Findings

The final consensus derived Cape Town ward MEWS observations chart incorporated seven physiological parameters with their respective colour-banded cut points (thresholds) and weighted trigger points (0 = normal, upper and lower 1 to 3).

### Strengths and Limitations of the Methods in Relation to Published Studies

To our knowledge this is the first study in South Africa to employ consensus methods and a multidisciplinary approach for the derivation of a local MEWS system for general hospital wards. One South African study reports the implementation of a UK Critical Care Outreach programme and MEWS in a public hospital in KwaZulu-Natal [34] but not the development of the chart.

The features of published consensus methods that enhanced respondents’ agreement included anonymity of private ranking, iteration by repeated rounds, controlled feedback and group response [24], [35], [36]. The Delphi method for data collection was inexpensive and convenient for respondents who had access to e-mail. The size of the Delphi group remained constant (n = 10) during the three rounds, which was likely to increase stability of the responses [37]. Conversely, the size of the face-to-face consensus group decreased from 11 participants in Round 1 to eight in Round 5, remaining within acceptable norms of eight to 11 [24]. One participant who contributed to only one face-to-face consensus meeting dropped out of the study.

The modified nominal group technique provided a structured environment in which experts were given the best available information [37] on MEWS. Face-to-face meetings between senior nurses and doctors opened up discussion about EWS and patient safety that may not otherwise have happened. Members’ varying levels of experience of a MEWS observations chart placed more responsibility on the facilitator than would otherwise have been the case [37]. The clinical responsibilities of the participants limited the time to less than two hours for each conference [38].

Construction of the Cape Town ward MEWS by consensus rather than cohort methods might suggest derivation of physiologic cut points and corresponding MEWS weighted trigger points based on clinical intuition. Difficulties relate to practical rather than theoretical considerations. To limit the possibility of best-guessing, the consensus group was presented with validated published MEWS (Table 1) as baseline values (33 cut points for seven physiological variables) but this may have influenced the final outcome.

### Strengths and Limitations of the Study Findings in Relation to Published Studies

Six of the Cape Town MEWS parameters (excluding urine output) are recommended for scoring by the Royal College of Physicians [13]. Although urine output is an early indicator of vascular compromise [39], [40] and was included in all six papers in a systematic review of track-and-trigger early warning systems [41], in one study urine output was missing in 97.1% (412/424) sets of observations [18]. For pragmatic reasons urine output measurement for the Cape Town ward MEWS was in millilitres (ml) of urine per hour [42] rather than volume per kilogram of body mass per hour [25] as not all patients are weighed on admission. The Cape Town ward MEWS chart did not require each patient’s ‘normal’ systolic BP as a baseline for interpreting MEWS as recommended in the literature [43], [44] and this is a limitation.

The GCS was incorporated into the Cape Town MEWS as an equivalent scale for the AVPU as alteration in mentation is reportedly an early sign frequently associated with SAEs [33]. Not all aggregate weighted track-and-trigger systems include the Glasgow Coma Scale (GCS) for grading level of consciousness, preferring the Alert/Responding to Voice/Responding to Pain/Unresponsive (AVPU) system because, although it may be possible to convert from GCS to AVPU, to convert from AVPU to GCS may not be possible [21]. The AVPU system is appropriate for monitoring mentation on a general ward but it was not intended for a specialist neurosurgical ward.

The algorithm for single parameters [45] and for aggregated MEWS systems [41] makes the chart a ‘combination’ track-and-trigger system. Similar systems operate in the UK [13], [46] and Australasia [47], [48]. The reason for the combined system is that aggregate scores may not trigger callout if only one variable falls outside the predetermined score, even though this has not been reported as a practical problem [49].

The local MEWS has a wider range of cut points for respiratory rate, heart rate, systolic BP and temperature than other published MEWS [15], [25], [42] and therefore possibly a more complicated response algorithm. By establishing local criteria for a MEWS in a single setting where the scale was developed [27], internal validity was increased, allowing for inferences to be drawn about the source population. No examples of consensus ranking sheets for the derivation of a MEWS system were found in the available literature.

It appears that clinical signs of deterioration (for example pallor, sweating, looking unwell) on the local MEWS chart are not often included in MEWS observations charts and were absent in the only example of a MEWS chart used in South Africa [34]. These variables require skills of observation, intuition, knowledge and experience for interpretation and may be as important as the physiological variables [6], [50], [51] as a MEWS system does not replace the clinical judgement of the nurse.

### Meaning of the Study: Possible Mechanisms and Implications for Clinicians or Policymakers

At the research setting the traditional ‘cardiac arrest team’ comprising a team of ICU nurses and doctors, had been replaced by individual ward response teams more than two decades previously. The individual ward response system, rather than centralized critical care outreach or acute care teams, risked lack of consistency in the recognition of and response to clinical deterioration. The MEWS reporting algorithm is intended to achieve decentralized consistency in the recognition of and response to clinical deterioration.

### Unanswered Questions or Future Research

Participants were from surgical wards. Cut points for each parameter may not be generalizable across broad diagnostic groups (respiratory disease, cardiac disease) and settings (medical wards, obstetrics, ICU, CCU, neurology).

Having more response algorithms might take clinicians longer to interpret and might delay a response but will have to be tested. Typically, ward patient monitoring responsibilities are delegated by registered professional nurses (RPNs) to registered staff nurses (RSNs) and nursing auxiliaries (RNAs), who may not have an appropriate level of scientific educational preparation to interpret signs of clinical and physiological deterioration.

Adverse events are also affected by clinical experience and professional education of nurses, nurse–patient ratios, and the environment but these factors were outside the scope of the study. For consensus methods more research is needed to clarify the concept ‘expertise’.

## Conclusion

The Cape Town ward MEWS observations chart was developed locally by consensus methods for bedside monitoring on general wards. Further research is needed to explore the ability of the MEWS chart to identify physiological and clinical deterioration.

## Supporting Information

Figure S1 **Example of an existing observations chart.**
(PSD)

Table S1 **Comparison of trigger thresholds for published and local MEWS parameters.**
(DOCX)
