# Supplementary material for: Monitoring Vital Signs: Development of a Modified Early Warning Scoring (Mews) System for General Wards in a Developing Country
Source: PLoS One. 2014 Jan 24;9(1):e87073. doi: 10.1371/journal.pone.0087073 (PMC3901724; doi:10.1371/journal.pone.0087073)
Supplement: Table S1 — Comparison of trigger thresholds for published and local MEWS parameters. (DOCX) [file pone.0087073.s002.docx]

Supporting Information 2

Comparison of trigger thresholds for published and local MEWS parameters

| Published MEWS | | | Local MEWS | |
| --- | --- | --- | --- | --- |
|  | Red trigger | Yellow trigger | Red trigger | Yellow trigger |
| Respiratory rate (breaths.min) | ≥30 | ≤9 or 21-29 | <8 or ≥30 | 8-9 or 21-29 |
| Heart rate (beats.min) | ≥130 | ≤40 or 111-129 | <40 or ≥130 | 40-50 or 111-129 |
| Oxygen saturation (%) | <85 | 85-89 | <85 | 85-89 |
| Systolic BP (mmHg) | ≤70 | 71-80 or >200 | ≤70 or >180 | 71-80 or 170-179 |
| Temperature (^o^C) | - | ≤35 or ≥38.5 | <34 or >39.6 | 34-35 or 38.6-39.5 |
| Neurological response | Unresponsive | Reacts to pain | Unresponsive | Reacts to pain |
| Urine output | 0.5 ml/kg/hr | - | >300 ml for 2 hrs or <20 ml/hr | ≤30 ml/hr |

BP, blood pressure
